# Monocytes from Cystic Fibrosis Patients Are Locked in an LPS Tolerance State: Down-Regulation of TREM-1 as Putative Underlying Mechanism

**DOI:** 10.1371/journal.pone.0002667

**Published:** 2008-07-16

**Authors:** Carlos del Fresno, Vanesa Gómez-Piña, Vanesa Lores, Alessandra Soares-Schanoski, Irene Fernández-Ruiz, Blas Rojo, Rodolfo Alvarez-Sala, Ernesto Caballero-Garrido, Felipe García, Tania Veliz, Francisco Arnalich, Pablo Fuentes-Prior, Francisco García-Río, Eduardo López-Collazo

**Affiliations:** 1 Research Unit, ‘La Paz’ Hospital, Madrid, Spain; 2 Service of Respiratory Diseases, ‘La Paz’ Hospital, Madrid, Spain; 3 Discover Unit, EMPIREO Molecular Diagnostic, Madrid, Spain; 4 Istituto Humanitas, Rozzano, Italy; 5 Emergency Service ‘La Paz’ Hospital and Department of Medicine, ‘La Paz’ Hospital Medical School, Universidad Autónoma de Madrid, Madrid, Spain; 6 Cardiovascular Research Center (CSIC-ICCC), Hospital de la Santa Creu i Sant Pau, Barcelona, Spain; University of Giessen Lung Center, Germany

## Abstract

Cystic Fibrosis (CF) is an inherited pleiotropic disease that results from abnormalities in the gene that codes for the chloride channel, Cystic Fibrosis Transmembrane Conductance Regulator (CFTR). CF patients are frequently colonized by several pathogens, but the mechanisms that allow colonization in spite of apparently functional immune systems are incompletely understood. In this paper we show that blood peripheral monocytes isolated from CF patients are found in an endotoxin tolerance state, yet this is not due to a deficient TLR activation. On the other hand, levels of the amplifier of inflammatory responses, TREM-1 (Triggering Receptor Expressed on Myeloid cells), are notably down-regulated in monocytes from patients, in comparison to those extracted from healthy volunteers. Furthermore, the soluble form of TREM-1 (sTREM-1) was not detected in the sera of patients. Additionally, and in strict contrast to patients who suffer from Chronic Obstructive Pulmonary Disease (COPD), CF monocytes challenged *ex vivo* with LPS neither up-regulated membrane-anchored TREM-1 nor sTREM-1. Finally, similar levels of PGE_2_ expression and p65 translocation into the nucleus were found in both patients and healthy volunteers, thus suggesting that TREM-1 regulation is neither controlled by PGE_2_ levels nor by p65 activation in this case. However, PU.1 translocation into the nucleus was significantly higher in CF monocytes than in controls, suggesting a role for this transcription factor in the control of TREM-1 expression. We conclude that down-regulation of TREM-1 expression in cystic fibrosis patients is at least partly responsible for the endotoxin tolerance state in which their monocytes are locked.

## Introduction

Cystic fibrosis (CF), also called mucoviscidosis, is a complex, pleiotropic disease that affects all exocrine epithelia. CF results from abnormalities in the gene that codes for the chloride channel termed CF Transmembrane Conductance Regulator (CFTR), which belongs to the extended family of ATP-binding cassette (ABC) transporter ATPases [1], [2]. This transmembrane glycoprotein is expressed in some epithelia, and controls chloride flux across cell surfaces. In addition, it down-regulates transepithelial sodium transport, regulates calcium-activated chloride channels and potassium channels, and may also serve important functions in exocytosis [1].

Some clinical features of CF include injuries of primary organs (pancreas, sinus, liver, intestine and exocrine pancreas) and secondary complications such as malnutrition, and diabetes. However, morbidity and mortality of CF patients are usually the result of chronic lower airway bacterial infections and inflammation of the lungs [3]. Repeated episodes of polymicrobial infection in these patients cause a progressive deterioration of lung tissue, a decline in pulmonary function and, ultimately, respiratory failure and death in 90% of CF patients [4]. In spite of the critical role played by pulmonary infections in these patients, the host factors that permit bacterial endobronchial colonization of the CF airway are poorly understood. It has been proposed that defective transmembrane conductance facilitates bacterial adherence to the respiratory epithelium, and that alterations in the sodium and chloride concentrations in the periciliary fluid impair the activity of defensins. Other authors claim that a deficit of hydrophilic surfactant proteins contributes to the inefficiency of the cellular inflammatory response to invading bacteria [5]. These observations, in turn, point to a deregulation of the innate immune systems in CF patients, and provoke a thorough analysis of the interplay between bacterial and host factors.

Recently, the immunoglobulin-related transmembrane receptor termed triggering receptor expressed on myeloid cells, (TREM)-1, has been recognized as an important element of the immune response, which strongly enhances leukocyte activation in the presence of microbial products [6], [7]. TREM-1 is mainly expressed on monocytes and neutrophils, and its levels at cell surface are up-regulated upon challenge with LPS or other microbial stimuli [6], [7], [8], [9]. Although the cellular signaling events downstream of TREM-1 engagement are incompletely understood, recently reported data suggest that receptor expression is essential for mounting an adequate inflammatory and cytotoxic response to polymicrobial sepsis [10]. This has been particularly demonstrated by *in vivo* silencing of *TREM-1* in a fecal peritonitis mouse model, which resulted in a blunted inflammatory response and increased mortality [7], [10], [11]. Also along these lines, receptor expression is significantly higher in the course of infectious diseases. Current evidence suggests that TREM-1 levels could represent a valuable marker of infection in several pathological conditions including sepsis [12], [13], [14]. LPS-induced receptor expression appears to be at least partly mediated by endogenous prostaglandin E2 (PGE_2_); this triggers EP4- and cAMP/protein kinase A-dependent mechanisms that are followed by p38 MAPK activation and PI3K-mediated signaling [13].

In addition to the membrane-bound form, a soluble TREM-1 variant (sTREM-1) has been detected in mouse and human serum [15], [16]. In particular, clinical studies have reported the presence of sTREM-1 in patients' serum and in bronchioalveolar lavage fluid [17], [18]. This soluble form has been found at particularly high concentrations in fluids from patients with microbial infections [7], [15], [19] and with ventilator-associated pneumonia [20]. Other authors also report an up-regulation of sTREM-1 in plasma from septic patients *versus* those suffering from systemic inflammatory response syndrome (SIRS) [19]. Altogether, current evidence strongly suggests an important role for sTREM-1 in the evolution of infectious diseases, and indicates that the soluble receptor form represents a useful marker of infection, particularly for the diagnosis of nosocomial sepsis [18] and pneumonia [18], [21], [22].

Two possible sources of sTREM-1 have been postulated: translation of an alternative TREM-1 mRNA splice variant and proteolytic cleavage (shedding) of mature, cell surface-anchored TREM-1 [7]. We have recently described that matrix metalloproteinases (MMPs) are responsible for shedding TREM-1 ectodomain from the cell membrane, most likely through proteolytic cleavage of a single peptide bond within the long juxtamembrane linker of the membrane-bound receptor [9]. These findings imply that sTREM-1 generation requires a previous expression of the membrane-anchored receptor.

Here we have tested the hypothesis that the high frequency of infection observed in CF patients is at least partly the consequence of deregulated innate immune responses. To this end, we have explored the expression of (1) receptors involved in LPS recognition (CD14 and TLR4/MD2), (2) the down-regulator of the TLR pathway, IRAK-M, and (3) both membrane-bound and soluble forms of TREM-1. We demonstrate that TREM-1 levels are clearly down-regulated in monocytes from patients when compared to healthy volunteers and to patients suffering from another pulmonary disease, COPD. Further, sTREM-1 was not detected in the sera of cystic fibrosis patients, and monocytes from these patients challenged *ex vivo* with LPS neither up-regulated TREM-1 nor its soluble form. On the basis of these findings, we conclude that polymicrobial lung infections in patients who suffer from cystic fibrosis are due at least partly to a deficiency in their innate immune responses, and that impaired TREM-1 expression plays a role in this deficiency.

## Materials and Methods

### Reagents

All reagents were of the highest quality available and were obtained from Merck (Darmstadt, Germany), Boehringer (Mannheim, Germany), or Sigma-Aldrich (St. Louis, MO). Lipopolysaccharide (LPS, from *Salmonella abortus*) was generously provided by Dr. Chris Galanos from the Max Planck Institut für Immunobiologie, Freiburg, Germany. The anti–TREM-1 and anti-TLR4 antibodies were purchased from R&D Systems (MN, USA); anti-CD14-PE, anti-CD16b and anti-CD1a were obtained from Serotec (Oxford, UK), anti-p65 from Santa Cruz (CA, USA), and anti-PU.1 from Cell Signaling (Danvers, MA).

### Patients and controls

We studied 20 non-smoker adults diagnosed with CF on the basis of established criteria (CFTR genotyping, sweat testing, and clinical phenotype) [23], who did not use inhaled or oral corticosteroids within the three months previous to the study. We also studied and ten consecutive clinically stable patients with moderate-severe COPD (post-bronchodilator FEV_1_<80% of predicted and FEV_1_/forced vital capacity (FVC)≤70%) [24]. Exclusion criteria included a history of asthma, other active lung disease, mental or physical handicap, or other significant diseases, such as congestive heart failure, ischemic or valvular cardiopathy or neuromuscular disease. None of the subjects had experienced an exacerbation or respiratory tract infection within the previous four weeks, and none of them showed significant bronchodilator reversibility (either >12% of baseline FEV_1_ or >200 ml). No subject has had oral corticosteroid therapy for at least three months. Moreover, ten sex- and age-matched healthy volunteers without neither personal history of CF nor other significant illness were also included as controls.

The following clinical variables were collected on each subject using chart review: age, sex, body mass index, CFTR genotype, dyspnea evaluated by the modified Medical Research Council (MRC) scale [25], arterial blood gases, basal spirometry, moderate-severe COPD exacerbations in the last year, COPD severity [26], microorganisms in sputum, and usual therapy. Blood samples were taken from patients and healthy donors. Peripheral blood mononuclear cells (PBMCs) were isolated and monocytes cultured under various conditions. Plasma from patients and healthy donors was also used for other assays (see below). Written informed consent was obtained from all subjects (patients and healthy volunteers). This study was approved by the local Ethics Committee (La Paz Hospital Ethics Committee, Paseo de La Castellana 261, Madrid 28046).

### Human monocytes isolation and culture

PBMCs were isolated from blood of healthy donors and CF patients by centrifugation on Ficoll-Hypaque Plus (Amershan Biosciences, Netherlands) following previously reported protocols [27], [28]. Cells were initially cultured for 2 h to a density of 10^6^ cells/ml in DMEN supplemented with antibiotics (100 IU/ml penicillin and 100 µg/ml streptomycin). After this period, the supernatant was removed and adherent cells (2×10^6^ per well) were cultured in the same medium supplemented with 10% heat-inactivated fetal bovine serum. Purity of all cultures was verified by CD14+ staining; on average 89% of the cells presented this surface marker. Cells were then cultured in the presence or absence of 10 ng/ml LPS for different periods of time (range: from 1 to 16 hours).

### FACS analysis of CD14, TLR4/MD2 and TREM-1 expression

Monocytes from healthy volunteers and patients were washed in PBS and incubated with anti-CD14, anti–TREM-1 or anti-TLR4/MD2 antibodies followed by an anti-goat FITC-conjugated secondary polyclonal antibody (Jackson Immunoresearch, Baltimore, USA). To correct unspecific binding, appropriate isotype control antibodies were used. For double staining (CD14 and TREM-1), cells were incubated with a CD14- allophycocyanin (APC)-conjugate (Miltenyi, Bergisch Gladbach, Germany). Samples were analyzed by flow cytometry using a BD FACSCalibur flow cytometer (BD Biosciences, San Diego, CA) equipped with a 25 mW argon laser. The same protocol was followed in cases of cultures stimulated with LPS.

### ELISA quantitation of sTREM-1

Concentrations of sTREM-1 in supernatants of human monocyte cultures from patients and controls were determined using a commercially available ELISA (DuoSet, R&D Systems), following the manufacturer's instructions (lower limit of detection: 15 pg/ml).

### Immunohistochemistry

Cells were fixed for 15 min with 2% paraformaldehyde in PBS, pH 7.3. Then, samples were blocked using a blocking serum (Universal Quick Kit, Vector Laboratories, CA), rinsed, and finally labeled by incubating overnight at 4 °C with anti–TREM-1 antibody diluted 1∶1,000 in PBS. The cells were washed twice in 20 mM Tris-HCl, pH 8.0, 150 mM NaCl, 0.2% Tween 20 (TTBS) and incubated with a secondary FITC-conjugated antibody (1∶4,000) for one hour. After rinsing with TTBS, cell nuclei were labeled by incubating with Hoechst 33342 (Molecular Probes), a cell-permeable fluorescent die that can therefore stain nuclei of both living and fixed cells. Briefly, Hoechst stock solution (2 mM in saline) was diluted in PBS (1∶8,000) and cells were incubated with this solution for 5–10 minutes at room temperature. Samples were then rinsed three times with PBS, excess buffer was drained, and samples finally mounted in anti-fade mounting medium. Cells were viewed and photographed on a Leica DMI6000 B (Leica Microsistemas, S.A.).

### RNA and protein isolation

Cells were washed once in PBS and their RNA was isolated using TRI-Reagent (IMICO, Cincinnati, OH). Purified RNA was treated with RNase-free DNase I (Amersham Biosciences) and cDNA was obtained by reverse transcription of 1 µg RNA using a poly(dT) oligonucleotide primer (Roche, Palo Alto, CA). Protein fractions were isolated as recommended (Roche), and the final pellets were dissolved in a solution containing 1% SDS.

### mRNA quantification

The expression levels of TNFα, IL-6, IRAK-M, and 18S were analyzed by real time Q-PCR (LightCycler; Roche Diagnostics, Indianapolis, IN), using cDNA obtained as described above. Real time Q-PCR was performed using a Fast-Start DNA master SYBR Green system (Roche) and specific primers (listed in Table 1). All results were normalized to the expression of the *18S* gene, and the cDNA copy number of each gene of interest was determined using a seven-point standard curve. Standard curves were run with each set of samples, the correlation coefficients r^2^ being >0.99. To confirm the specificity of the reaction products in each experiment, the melting profile of each sample was analyzed using the LightCycler. Melting profiles were determined by maintaining the reactions at 80°C for 10 s and then increasing the temperature to 95°C at a linear rate of 0.1°C/s while measuring the emitted fluorescence. Analysis of the melting curves demonstrated that each pair of primers amplified a single product. PCR products were separated in agarose gels and stained with ethidium bromide to confirm that in each case a single fragment of the predicted size had been amplified. For 18S mRNA detection we used the primers of QuantumRNA Classic 18S provided by Ambion, USA. All other primers were purchased from IZASA (Barcelona, Spain) and their specific PCR conditions are listed en Table 2.

**Table 1:** Sequences of oligonucleotides used for Q-PCR analysis.

Gene	Sense primer (3′→5′)	Antisense primer (3′→5′)
*TNFα*	GCCTCTTCTCCTTCCTGATCGT	CTCGGCAAAGTCGAGATAGTCG
*IL-6*	CAAAGAATCTAGATGCAATAA	GCCCATTAACAACAACAATCTG
*IRAK-M*	TTTGAATGCAGCCAGTCTGA	GCATTGCTTATGGAGCCAAT

**Table 2:** PCR conditions for mRNA quantitation.

Gene	Tm (°C)	Cycles
*TNFα*	64	95°C for 10 s, 64°C for 10 s, 72°C for 19 s
*IL-6*	50	95°C for 10 s, 50°C for 10 s, 72°C for 10 s
*IRAK-M*	59	95°C for 10 s; 59°C for 10 s 72°C for 19 s

### Isolation of nuclear proteins

A procedure based on the method reported by Lopez-Collazo *et al* [29] was used. Cells were washed twice with ice-cold PBS, and plates were filled with 0.4 ml Buffer A (20 mM Tris-HCl, pH 7.8, 5 mM MgCl_2_, 10 mM KCl, 0.5 mM EGTA, 0.5 mM DTT, 1 mM PMSF, and 10 µM leupeptin). The cells were scraped off the dishes using a rubber policeman and transferred to 1.5-ml Eppendorf tubes, to which Nonidet P-40 was added to reach a 0.5% final concentration. The tubes were gently vortexed for 15 s and nuclei were sedimented by centrifugation at 8,000×g for 15 s. Aliquots of the supernatant were stored at −80°C (cytosolic extract), and the nuclear pellet was resuspended in 100 µl Buffer A supplemented with 0.4 M KCl. Nuclear proteins were extracted by centrifugation at 13,000×g for 15 min, and aliquots of the supernatant were stored at −80°C. All steps of cell fractionation were conducted at 4°C.

### SDS-PAGE and Western blotting

Cell extracts and nuclear proteins were boiled in Laemmli buffer, resolved on 15% SDS–polyacrylamide gels in Tris/glycine/SDS buffer, and transferred to Immun-Blot PVDF Membranes (Bio-Rad, CA) at 300 mA for 1.5 h at 4°C. After blocking for 1 h in TTBS containing 5% non-fat milk, membranes were washed three times in TTBS alone and probed for 20 h with anti–TREM-1, anti-p65 or anti-PU.1 antibodies diluted in TTBS. Following extensive washing in TTBS, membranes were incubated for 45 min with a secondary HRP-conjugated antibody (diluted 1∶4,000), and finally washed three times with TTBS. The bound antibodies were detected using ECL Plus reagents according to the manufacturer's instructions (Amersham–Pharmacia Biotech, Holland).

### Prostaglandin E2 quantitation

Levels of PGE_2_ in sera and supernatants of cultures of monocytes from CF patients and healthy controls were measured using a commercial available kit (R&D Systems) and following the manufacturer's instructions.

### Data analysis

The number of experiments analyzed is indicated in each figure. The statistical significance was calculated using the unpaired Student's test and differences were considered significant at p values<0.05.

## Results

### Patients

Clinical and genetic data of CF patients included in the current study (n = 20) are shown in Table 3. Their mean age was 30±10 years (mean±SD); their FEV_1_ (% predicted) was 61±21%, their FEV_1_/FVC ratio 69±102% and the mean incidence of pulmonary exacerbations in the previous year was 1.8 episodes (range, 0–5). In agreement with the genetic distribution in the Spanish population, nine patients (45%) were homozygous for the CFTR variant ΔF508 and other nine were compound heterozygotes, with one copy of ΔF508 plus another mutation. *Pseudomonas aeruginosa* colonization was identified in 14 CF patients (70%).

**Table 3:** Clinical data of cystic fibrosis patients included in the current study. MAC, *Mycobacterium avium* complex.

Subject	Age (yr)	Gender	BMI (Kg/m^2^)	CFTR mutation	FEV_1_ (%)	FEV_1_/FVC(%)	Microorganisms in sputum	Inhaled bronchodilator	Human DNase?	Inhaled antibiotics
1	23	M	20.2	ΔF508 / ?	59.5	55.9	*P. aeruginosa*	Formoterol	No	Colimycin
2	39	M	25.1	ΔF508 / ΔF508	87.4	81.4	*P. aeruginosa*	Salbutamol	2.5 mg/d	Tobramycin
3	28	F	20.1	ΔF508 / ?	66.2	82.2	*H. influenzae*, *S. aureus*	Formoterol	No	None
4	31	M	17.7	ΔF508 / ΔF508	21.6	48.8	*C. albicans*	Salmeterol, salbutamol	2.5 mg/d	None
5	23	F	18.3	ΔF508 / W1282x	76.3	88.8	*H. influenzae*, *S. aureus*, *P. aeruginosa*	Formoterol	2.5 mg/d	Tobramycin
6	30	F	21.8	ΔF508 / ?	32.8	65.3	*P. aeruginosa*	Salbutamol, ipratropium	No	Colimycin
7	30	M	25.2	ΔF508 / ΔF508	73.8	70.6	*P. aeruginosa*	Salmeterol	No	Tobramycin
8	28	F	18.5	ΔF508 / ?	80.1	75.2	*H. influenzae*, *S. aureus*, *MAC*	Salbutamol	No	None
9	28	M	22.3	ΔF508 / ?	81.0	79.1	*B. cepacia*	None	No	None
10	21	M	23.2	*R553 / 2789+5G->A*	85.2	72.9	*S. aureus*, *P. aeruginosa*	None	No	Tobramycin
11	29	M	21.8	ΔF508 / ΔF508	33.9	48.4	*P. aeruginosa*	Salmeterol, salbutamol	No	Colimycin
12	68	F	26.3	ΔF508 / ΔF508	65.0	70.3	*P. aeruginosa*	Salmeterol	No	None
13	32	M	22.8	ΔF508 / ΔF508	79.4	75.1	*P. aeruginosa*	Salbutamol	No	None
14	22	F	23.0	ΔF508 / ΔF508	35.9	55.4	*S. aureus*, *P. aeruginosa*	Formoterol, salbutamol	2.5 mg/d	Tobramycin
15	27	F	20.8	R334W / 1069delICA	47.5	66.2	*P. aeruginosa*	Salmeterol, salbutamol	No	Tobramycin
16	26	M	21.1	ΔF508 / ?	82.4	76.2	*H. influenzae*, *S. aureus*, *P. aeruginosa*	None	No	None
17	31	M	22.4	ΔF508 / ΔF508	51.2	64.3	*P. aeruginosa*	Salmeterol	No	Tobramycin
18	24	M	21.7	ΔF508 / ?	83.2	71.7	*H. influenzae*, *S. aureus*	None	No	None
19	23	F	20.3	ΔF508 / ΔF508	43.1	65.9	*P. aeruginosa*	Formoterol	No	Colimycin
20	28	F	23.7	ΔF508 / ?	39.8	67.0	*P. aeruginosa*	Salbutamol, salmeterol	No	Tobramycin

Mean age of moderate-severe COPD patients was 69±7 years, with a mean post-bronchodilator FEV_1_ of 53±20% predicted and a FEV_1_/FVC ratio of 45±12% (Table 4). The incidence of COPD exacerbations (2.2 episodes/year) was similar to that of the CF patients. All patients were informed of our study. This study was approved by the local Ethics Committee (La Paz Hospital Ethics Committee, Paseo de La Castellana 261, Madrid 28046).

**Table 4:** Clinical data of Chronic Obstructive Pulmonary Disease patients included in the current study. NAC, N-Acetyl-Cystein, …

Subject	Gender	Age (yr)	BMI (Kg/m^2^)	FVC (%)	FEV_1_ (%)	FEV_1_ /FVC	PaO_2_ (mmHg)	PaCO_2_ (mmHg)	MRC	Exacerbations last year / Grade	Treatment
1	M	82	23.6	109	73	0.49	55.8	41.6	4	3 / IV	LTOT, salmeterol, fluticasone (2000 mcg), ipratropium, NAC, salbutamol
2	M	69	29.1	77	33	0.33	67.5	42.8	2	4 / III	Salmeterol, fluticasone (1000 mcg), tiotropium, salbutamol, NAC
3	M	68	25.2	59	21	0.27	68.8	48.4	3	2 (3) / IV	Salmeterol, fluticasone (1000 mcg), tiotropium, teophiline, NAC, salbutamol
4	M	68	23.5	100	44	0.35	78.0	37.3	2	0 / III	Tiotropium, salbutamol
5	M	65	22.9	77	35	0.35	60.5	55.5	2	1 / III	Formoterol, budesonide (800 mcg), tiotropium, salbutamol
6	M	73	28.0	69	43	0.48	56.1	46.1	2	2 / III	Salmeterol, fluticasone (2000 mcg), tiotropium, salbutamol
7	F	66	30.5	133	75	0.47	73.0	35.2	1	3 / II	Salmeterol, fluticasone (1000 mcg), tiotropium, salbutamol
8	M	59	28.7	84	67	0.63	78.6	34.6	2	2 / II	Tiotropium, salbutamol
9	M	79	29.3	90	68	0.56	69.9	32.0	2	0 / II	Salmeterol, ipratropium
10	M	63	34.8	92	69	0.59	58.3	38.6	4	5 / II	Salmeterol, fluticasone (1000 mcg), ipratropium, teophilline, NAC, salbutamol

### Circulating monocytes from cystic fibrosis patients fail to mount an appropriate inflammatory response in the presence of Gram-negative endotoxin

**Figure 1:**
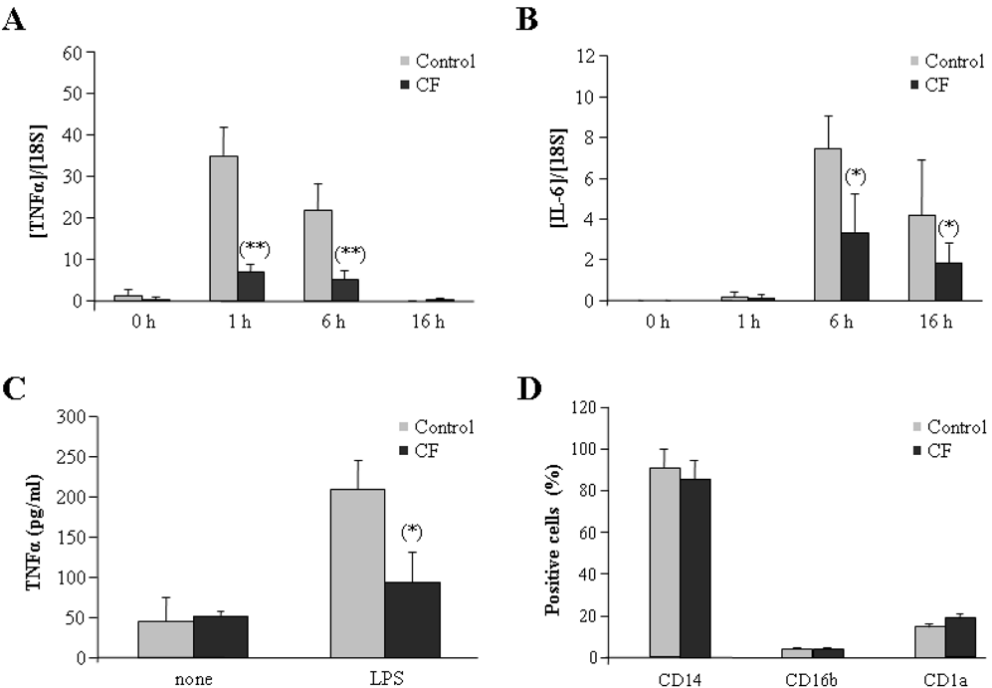
Inflammatory responses are impaired in monocytes from CF patients. Monocytes from healthy volunteers (control, gray bars, n = 10) and from CF patients (CF, solid bars, n = 20) were cultured in the presence of 10 ng/ml LPS for indicated times. Cells were harvested, total RNA isolated and cDNA synthesized. TNFα (A) and IL-6 (B) mRNA levels were analyzed by real time Q-PCR. The ratios [TNFα]/[18S] and [IL-6]/[18S] are depicted. **, p<0.01 with respect to healthy controls (C) Concentrations of TNFα were determined in supernatants of cultures of human monocytes from healthy volunteers (control, gray bars, n = 10) and CF patients (CF, solid bars, n = 20), stimulated or not with 10 ng/ml LPS. *, p<0.05 with respect to healthy controls. (D) Monocytes isolated from healthy volunteers (control, gray bars, n = 10) and CF patients (CF, solid bars, n = 20) were stained with anti-CD14-PE, anti-CD16b or anti-CD1a antibodies and then analyzed by flow cytometry; the fraction of cells stained with each antibody is given.

Because CF patients are frequently colonized by different bacteria [30], [31], we decided to study innate immune responses in circulating monocytes isolated from these patients. First, we cultured these cells in the presence or absence of LPS for 1, 6 or 16 hours, and verified production of pro-inflammatory cytokines. This analysis revealed low TNFα and IL-6 mRNA expression levels in monocytes isolated from CF patients (in following termed CF monocytes), while cells from healthy volunteers were able to induce significant amounts of these two cytokines after LPS challenge (Fig. 1, A and B). We note in particular that one-hour LPS treatment was enough to induce a clear expression of TNFα mRNA, which remained high for at least six hours. By contrast, CF monocytes failed to potently up-regulate this cytokine. Similar results were observed with IL-6 levels, but maximal expression in healthy volunteers was observed 6 h after LPS challenge, and the difference between normal and CF monocytes was not so pronounced.

In addition, we also studied production of soluble TNFα in the supernatants of these cultures. In line with our findings regarding mRNA transcription, high levels of TNFα protein were detected 16 hours after LPS stimulation in monocytes isolated from healthy volunteers. By contrast, cells from CF patients produced about 2.5-times less TNFα than controls (Fig. 1C). Thus, we conclude that CF monocytes fail to mount a strong innate immune response upon LPS challenge. We note that all monocyte cultures were reasonably pure, as illustrated by the presence of an average of 89% CD14-positive cells in both healthy controls and CF patients, with negligible levels of CD16b and CD1a staining (Fig. 1D).

### Circulating monocytes from CF patients exhibit normal levels of TLR4 and MD2 at their cell surface, and do not overexpress IRAK-M

**Figure 2:**
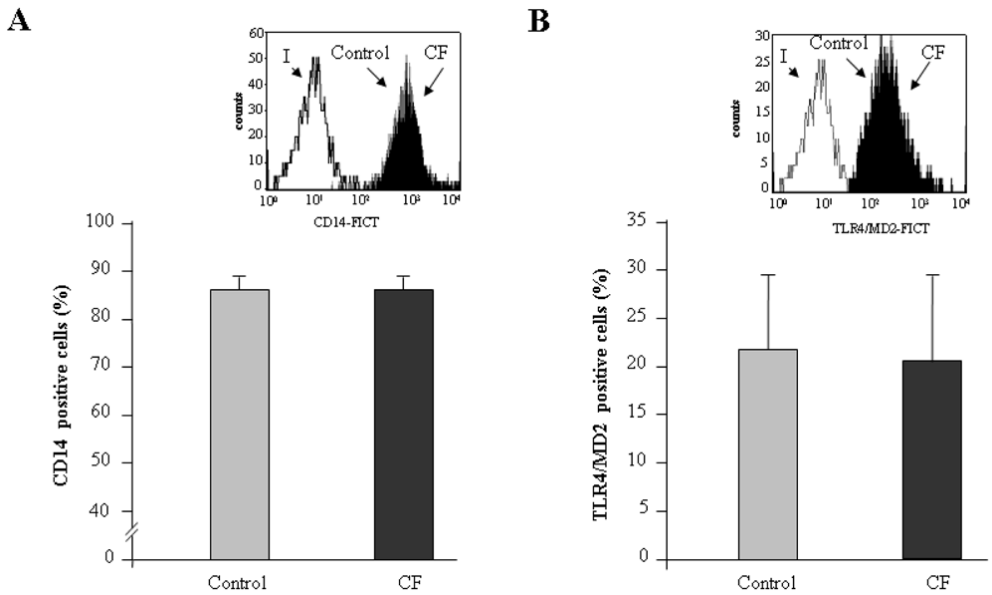
Elements of the LPS receptor complex are expressed at similar levels in control and CF monocytes. Monocytes isolated from healthy volunteers (control, gray bars, n = 10) and CF patients (CF, solid bars, n = 20) were stained with anti-CD14 and anti-TLR4/MD2 and then analyzed by flow cytometry; the fraction of cells stained is given (A and B respectively). Typical histograms are shown in the insets (I, isotype; Control, healthy volunteers; CF, Cystic Fibrosis).

Once established that CF monocytes fail to respond properly to *ex vivo* LPS challenge, and having observed similar levels of CD14 expression in these cells and in control monocytes (Figs 1D and 2A), we decided to analyze the expression of the two other proteins involved in LPS recognition and signaling, MD2 and TLR4. It is well known that this Toll-like receptor together with CD14 and the soluble carrier, MD2, form the receptor complex for LPS in human monocytes [32]. Thus, abnormalities in TLR4 and/or MD2 expression could offer a straightforward explanation for the LPS desensitization phenomenon observed in CF monocytes. However, and in contrast to our expectations, normal levels of these two proteins were found at the cell surface, and the flow cytometric analysis of TLR4/MD2 expression did not show significant differences between healthy controls and CF patients in this regard (Fig. 2B).

**Figure 3:**
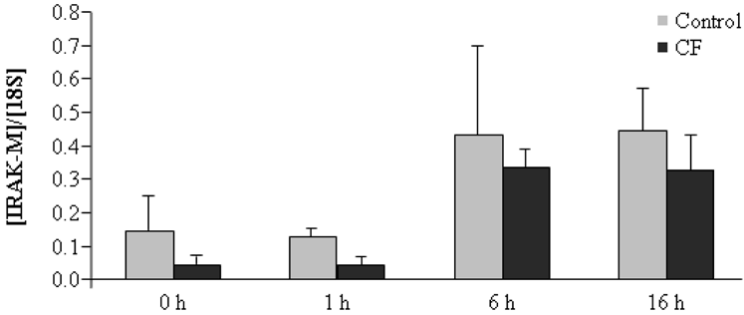
IRAK-M is normally induced in CF monocytes after LPS stimulation. Cultures of human monocytes from healthy volunteers (control, gray bars, n = 10) and CF patients (CF, solid bars, n = 20) were treated with 10 ng/ml LPS for indicated times. Next, cells were harvested, total RNA isolated and cDNA synthesized as described in Material and Methods. The ratio of IRAK-M to 18S is shown.

We have previously described that the pseudokinase, IRAK-M, is overexpressed in monocytes from patients who suffer from Acute Coronary Syndrome, and is involved in the endotoxin tolerance phenomenon observed in these monocytes [33]. Next, and considering this negative role within the TLR signaling pathway, we determined IRAK-M expression levels in LPS-stimulated monocytes [27], [34], [35]. However, despite a slight tendency to IRAK-M down-regulation in CF monocytes compared to controls, no significant differences were observed in the kinetics of expression of this pseudokinase (Fig. 3).

### TREM-1 is expressed at low levels in circulating monocytes from cystic fibrosis patients

Impaired up-regulation of *TNFα* and *IL-6* (Fig. 1) would suggest that CF monocytes are not able to mount a strong inflammatory response after LPS challenge. This defective response to endotoxin is most likely not due to impaired LPS recognition by the MD2-CD14-TLR4 complex, as the three proteins are normally expressed in these cells (Fig. 2), nor to IRAK-M overexpression (Fig. 3). Therefore, we reasoned that a downstream positive feed-back loop could be affected in CF monocytes. Because of its repeated association with polymicrobial infections, and in particular with lung infections [17], [36], [37], [38], we hypothesized that decreased expression of another receptor involved in inflammatory responses, TREM-1, could lead to the observed LPS unresponsiveness in patients monocytes, with concomitant impairment of inflammatory responses. Although the actual role of TREM-1 in the innate immune response is not well understood, current evidence strongly supports a key role for this receptor as an amplifier of inflammatory responses, in particular during polymicrobial sepsis (see Introduction and refs [6], [39]).

**Figure 4:**
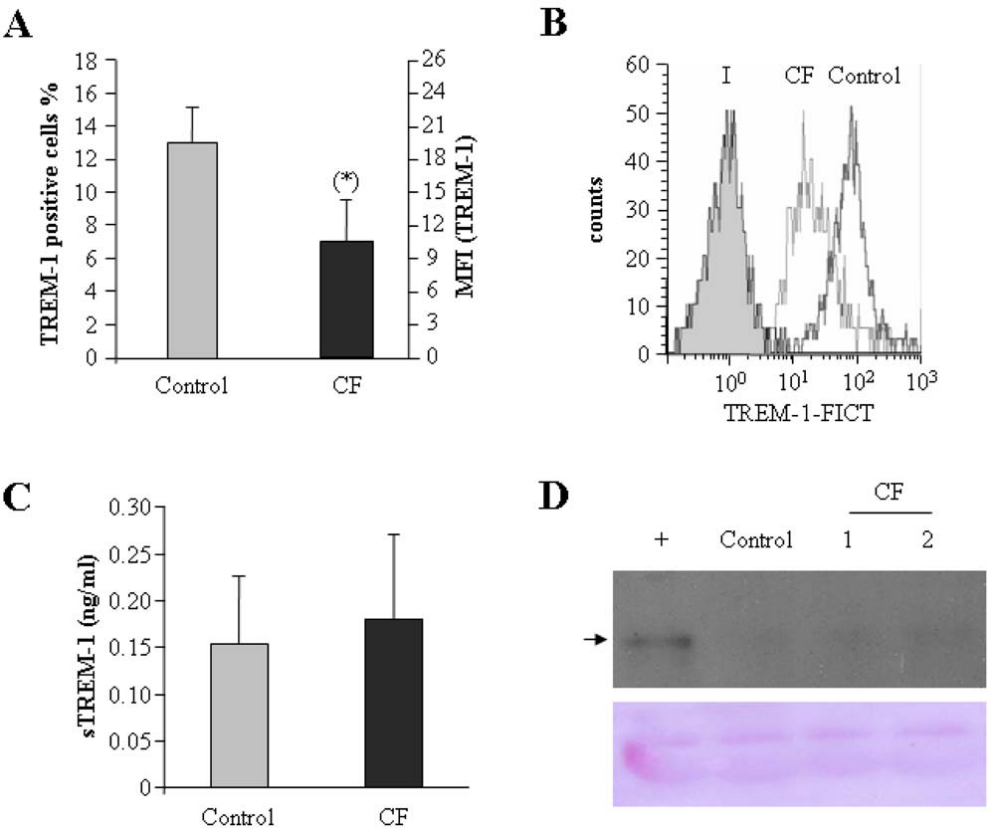
Expression levels of TREM-1 and sTREM-1 are low in CF monocytes. (A) Monocytes isolated from healthy volunteers (controls, gray bars, n = 10) and CF patients (CF, solid bars, n = 20) were stained with anti–TREM-1-FITC and then analyzed by flow cytometry; the fraction of cells stained is given as % of total cells (left scale) and as mean fluorescence intensity (MFI, right scale). *, p<0.05 with respect to healthy controls. (B) A typical histogram obtained from flow cytometric analysis of TREM-1 expression is shown (I, isotype; CF, CF patients; Control, healthy volunteers). (C) The concentration of sTREM-1 in the supernatants of the same cultures was analyzed with a commercial ELISA. The mean concentration of sTREM-1 is represented. (D) Supernatants from monocyte cultures (one from a healthy volunteer (control) and two from randomly selected CF patients) were centrifuged at 400×g to remove detached cells and possible cellular debris. A Western blot analysis of these supernatants was performed using an anti–TREM-1 commercial antibody that recognizes the extra-cellular domain of TREM-1. Human monocytes challenged with LPS for six hours were used as positive control (+). Notice detection of a band of approximately 27 kDa in this condition (marked with an arrow). Loading controls are also shown (membrane stained with Ponceau red, lower panel). The results of a triplicated experiment are shown.

To verify this hypothesis, we compared TREM-1 levels in monocytes isolated from CF patients versus healthy volunteers. Indeed, a significant down-regulation of membrane-anchored TREM-1 was observed in CF monocytes (Fig. 4, A and B). These findings indicate that basal levels of TREM-1 are about two-fold lower in circulating monocytes from CF patients than in healthy individuals, and suggest a possible explanation for the observed inability of these cells to mount an inflammatory response.

We have recently reported that expression levels of TREM-1 at the monocyte surface are controlled by endogenous metalloproteinases, which cleave membrane-anchored TREM-1 to generate the soluble form, sTREM-1 [9]. High levels of soluble TREM-1 have been identified by others in patients suffering from diverse infectious diseases [8], [16] and in particular in their fluids [14], [15], [17], [18], [19], [20]. Our previous findings suggest that LPS first induces an appreciable up-regulation of TREM-1 at the cell surface of human monocytes, but this membrane-anchored form is subsequently downregulated due to enhanced metalloproteinase production and/or activation, which generates sTREM-1 via proteolytic cleavage of the full-length receptor [9]. Thus, these clear differences in TREM-1 levels at the monocyte surface could be explained by the presence of a pathogen infection in CF patients. In particular, LPS generated by Gram-negative pathogens could induce metalloproteinase expression and activation, and concomitant shedding of TREM-1 extracellular domain. To test this possibility we compared the levels of sTREM-1 in sera from CF patients vs. healthy controls. However, no significant differences were found between both groups, and levels of sTREM-1 were low (<0.2 ng/ml) in both CF patients and healthy volunteers (Fig. 4C). These ELISA data were further confirmed via Western blot analysis (Fig. 4D).

### Circulating monocytes from CF but not COPD patients fail to overexpress TREM-1 at the cell surface upon LPS stimulation

**Figure 5:**
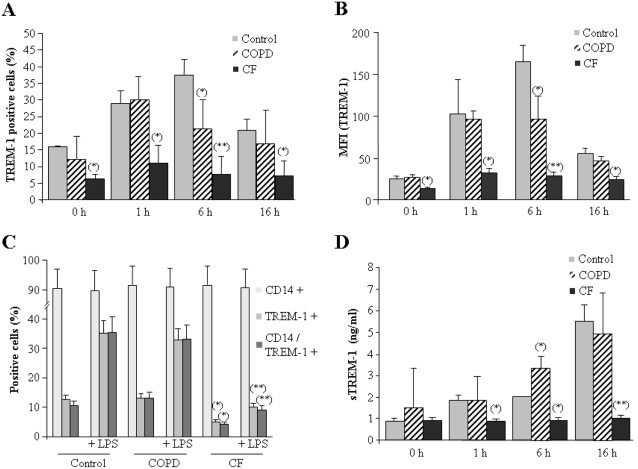
CF but not COPD monocytes fail to induce TREM-1 expression after LPS stimulation. (A) Cultures of human monocytes from healthy volunteers (control, gray bars, n = 10) CF patients (CF, solid bars, n = 20) and COPD patients (COPD, hatched bars, n = 10) were treated with 10 ng/ml LPS for indicated times. Next, cells were harvested and stained with anti-TREM-1-FITC and analyzed by flow cytometry. The fraction of positive cells is given as % of total cells (A) and MFIs (B). The percentage of CD14 and TREM-1 positive cells was analyzed in monocytes from five of each of the studied groups (i.e., CF patients, COPD patients and healthy individuals; all samples were randomly selected), treated or not *ex vivo* with LPS for 1 hour. The flow cytometry analysis of these cells simultaneously stained with CD14-APC and TREM-1-FITC is shown (C). Supernatants from the same experiment (shown in panels A and B) were centrifuged at 400×g to remove detached cells and possible cellular debris. The concentration of sTREM-1 in the supernatants was analyzed with a commercial ELISA. The mean concentration of sTREM-1 in ng/ml is given (D). *, p<0.05 and **, p<0.01 with respect to similarly treated monocytes from healthy individuals.

Previous studies have demonstrated a fast up-regulation of membrane-anchored TREM-1 expression after LPS challenge [7], [8]. Considering our previous findings, we next analyzed levels of TREM-1 in CF monocytes stimulated with LPS. As we have shown before [9], a time course analysis of TREM-1 expression revealed that levels of this receptor increase in a time-dependent manner in monocytes isolated from healthy controls, reaching a maximum (three-fold increase over basal levels) about six hours after LPS challenge (Fig. 5A and B, gray bars). TREM-1 expression increased to similar levels in COPD monocytes 1 h after stimulation, but appeared to reach a maximum earlier than in control monocytes (Fig. 5A and B, hatched bars). In striking contrast, receptor expression was not up-regulated in CF monocytes upon *ex vivo* endotoxin stimulation (Fig. 5A and B, solid bars). In addition, and to exclude that the observed effect might be due to a reduction in the number of TREM-1 positive cells, we performed another experiment with monocytes isolated from five of each of the three groups studied (randomly selected from the previously discussed sets). Cultures of circulating monocytes were stimulated, or not, with LPS for 1 hour. Next, cells were double-stained with TREM-1-FITC and CD14-APC, and analyzed by flow cytometry. As figure 5C shows, about 90% of cells were CD14+ in all conditions analyzed. Control cells stimulated with LPS exhibit an average of 40% CD14+/TREM-1 positive cells, and a similar result was obtained for COPD monocytes. In contrast, CF cells exhibit low levels of CD14+/TREM-1+ cells (less than 5%), and they did not increase significantly their basal levels of TREM-1 after LPS stimulation. No significant cell viability changes were detected in both CF and COPD monocytes (data not shown).

We also verified that sTREM-1 is not detected in supernatants of untreated cultures of human monocytes isolated from healthy volunteers, which implies levels below 15 pg/ml. However, concentration of this soluble form was notably enhanced in the supernatants of both control and COPD cultures after 16 hours of LPS stimulation (Fig. 5D). In our model, reduced cell surface expression of TREM-1 was always accompanied by a concomitant increase in sTREM-1 levels in culture supernatants. By contrast, cultures of CF monocytes did not contain detectable levels of sTREM-1 after LPS stimulation (Fig. 5D, solid bars).

**Figure 6:**
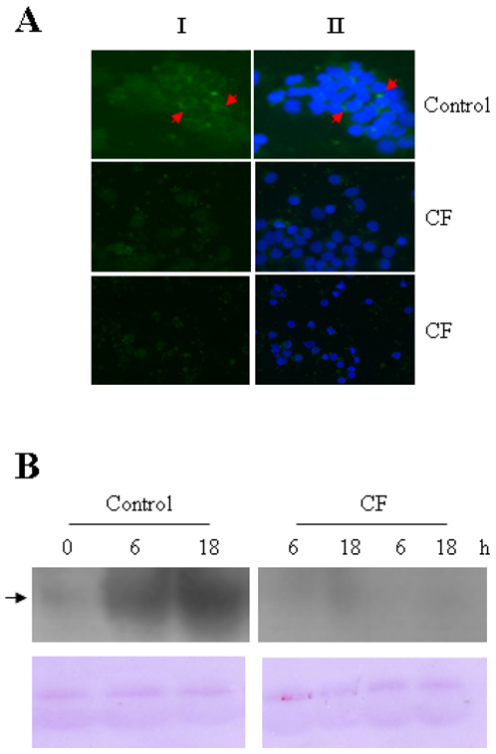
TREM-1 is not induced in CF monocytes upon LPS challenge. (A) Cultures of human monocytes from healthy volunteers and CF patients were treated with 10 ng/ml LPS for 6 hours, stained with an anti–TREM-1 antibody (green), and nuclei were localized via standard staining with Hoechst 33342 (blue). A typical result of three independent experiments is shown (I, anti-TREM-1; II, merged anti-TREM-1 and Hoechst 33342). (B) Cell supernatants from monocyte cultures (one from a healthy volunteer (control) and two from randomly selected CF patients) were stimulated with LPS or not (0) for 6 and 18 h, and then centrifuged at 400×g to remove detached cells and possible cellular debris. A Western blot analysis of these supernatants was performed using an anti–TREM-1 commercial antibody that recognizes the extracellular domain of the receptor. Notice detection of a band of approximately 27 kDa in this condition (arrow). Loading controls are also shown (membrane stained with Ponceau red, lower panel). The results of a typical experiment from three experiments performed are shown.

We further confirmed these results by immunofluorescence and Western blot analysis. As shown in Figure 6A, significant TREM-1 expression is observed in cultures of human monocytes treated with LPS for 6 h but not in those cells isolated from CF patients. In addition, these experiments clearly demonstrate membrane localization of mature TREM-1. On the other hand, the absence of sTREM-1 in the supernatant of CF cultures was confirmed by Western blotting (Fig. 6B).

### Levels of PGE_2_ do not account for TREM-1 down-regulation in CF monocytes

**Figure 7:**
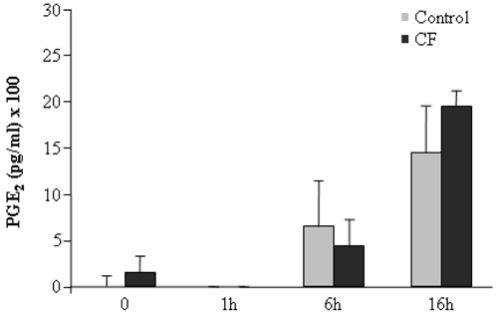
Levels of PGE_2_ are similar in CF monocytes and controls. Cultures of human monocytes from healthy volunteers (control, gray bars, n = 10) and CF patients (CF, solid bars, n = 20) were treated with 10 ng/ml LPS for indicated hours. Next, levels of PGE_2_ were analyzed using a PGE_2_ EIA kit.

It has been recently demonstrated that prostaglandin E2 (PGE_2_) plays an important role in the control of TREM-1 expression [13]. In particular, Murakami and coworkers suggest that LPS-induced TREM-1 expression on macrophages is mediated by endogenous PGE_2_. To examine possible mechanisms underlying TREM-1 down-regulation in CF patients, we determined levels of PEG_2_ in monocytes from CF and healthy volunteers. However, and contrary to expectations, there were no significant differences in PGE_2_ concentrations between CF and control monocytes (Fig. 7). This finding indicates that other factors in addition to PEG_2_ modulate TREM-1 expression in human monocytes.

### High levels of nuclear PU.1 in circulating monocytes from cystic fibrosis patients

**Figure 8:**
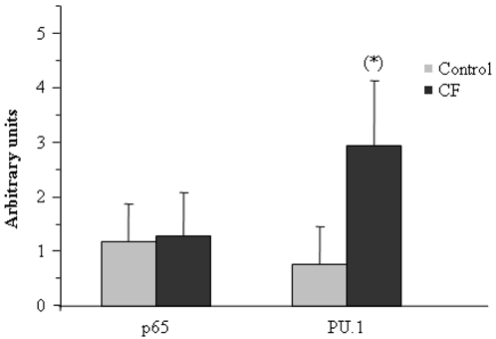
Levels of PU.1 but not of p65 are up-regulated in CF monocytes. Nuclear protein fractions were isolated from circulating monocytes obtained from healthy volunteers (control, gray bars, n = 5) and CF patients (CF, solid bars, n = 10). Western blot analysis was performed using anti-p65 and anti-PU.1 antibodies. Normalization was carried out with an anti-α-actin antibody. The average of densitometer analysis of each Western blot is shown (n = 5). *, p<0.05 with respect to healthy controls.

It has been recently reported that activation of the transcription factor p65 is necessary for expression of TREM-1, whereas another transcription factor, PU.1, inhibits TREM-1 up-regulation [40]. PU.1 is highly expressed in cells of the immune system and regulates several genes in, e.g., macrophages and B cells [41]. Apparently, in response to LPS challenge both factors bind to the *TREM-1* promoter in macrophages. To analyze possible mechanisms underlying TREM-1 down-regulation in CF patients, we determined levels of p65 and PU.1 in nuclei isolated from CF monocytes and healthy volunteers. As Figure 8 shows, PU.1 but not p65 is differentially activated in untreated monocytes from CF patients versus controls. These findings suggest that PU.1 activation plays an important role in the mechanism leading to TREM-1 down-regulation in cystic fibrosis patients.

## Discussion

Although previous investigations have revealed a hyperinflammatory state in CF patients [42], [43], [44], the observed high frequency of pathogen colonization in these patients points to a significant deficiency of their innate immune systems. Several studies conducted so far have focused on local and resident cells (e.g., lung epithelial cells or recruited neutrophils), and have demonstrated a defective IL-8 secretion by CF-airway epithelial cells in these patients [45]. On the other hand, a recently published article presents an interesting mechanism by which neutrophils fail to be activated in CF patients' lungs [46]. The unopposed elastolytic activity in the airways of individuals with CF leads to CXCR1 cleavage on neutrophils, thus disabling their bacterial-killing capacity.

In contrast, the inflammatory responses of circulating cells in CF patients have not been properly described so far. Previous works indicate that levels of TNFα in CF patients could be either higher [42] or lower than in healthy volunteers [43]. These differences appear to be due to the presence of *P. aeruginosa* infection and/or antibiotic treatment, but the small number of enrolled patients (14 and 10, respectively) does not allow for a statistically meaningful comparison. Here we show that monocytes isolated from twenty CF patients, fourteen of which were colonized by *P. aeruginosa* (see Table 3), are unable to mount a proper inflammatory response when they are challenged *ex vivo* with the major immunogenic component of Gram-negative bacteria, LPS. Besides, this feature is irrespective of the presence or absence of *P. aeruginosa* infection. The low levels of two critical pro-inflammatory cytokines, TNF-α and IL-6, produced by these cells indicate that they could not properly respond to a bacterial insult (Figure 1). Our findings are in agreement with the significantly lower IL-8 production in ΔF508 CF cell line compared to a normal airway epithelial cells after LPS challenge [47], but are at odds with the higher levels of TNFα expression in CF monocytes stimulated with LPS recently reported by others [44]. This discrepancy might result from the use of a 10-fold higher LPS concentration by Jarešová and co-workers. Besides, only nine patients were included in the latter study. Additional studies of immune responses by CF monocytes enrolling a larger number of patients will be needed to gain further insight into the mechanisms that ultimately result in chronic lung colonization in these patients.

Our current results suggest that the observed refractory state in CF patients was not secondary to impaired LPS recognition by circulating CF monocytes. Indeed, we observed normal levels of TLR4, MD2 and CD14 in cells from CF patients and healthy individuals; similar observations were made for other elements of the intracellular TLR signaling pathway. These data are in line with those reported by Muir and coworkers, who showed that the expression of TLRs is not directly affected by CFTR dysfunction in different types of airway epithelial cells [47].

Note that in our experiments we have used a purified lipopolysaccharide from *Salmonella abortus* generously provided by Dr. Chris Galanos (Max Planck, Freiburg, Germany). Recently, a thorough study that compared endotoxins from different sources indicated that LPS from *S. abortus* is more potent than the one generated by the bacteria most commonly found in CF patients, *P. aeruginosa* [48]. Despite this, *ex vivo* stimulation of CF monocytes with *S. abortus* LPS did not generate the pro-inflammatory response observed in cells from healthy controls. It is therefore safe to assume that challenging CF monocytes with P. *aeruginosa* LPS would lead to similar or even less pronounced responses.

Several recent reports demonstrate an important role for TREM-1 in inflammation during an infectious disease; the receptor appears to act as an amplifier of the immune response in the presence of pathogens [6], [11], [12], [17], [39]. In line with these findings, we observed that circulating monocytes from patients who suffer from CF showed significantly decreased basal levels of TREM-1. In addition, the receptor was not up-regulated upon LPS stimulation *ex vivo*. These observations suggest that down-regulation of surface exposed TREM-1 might at least partly underlie the non-responsiveness state in CF patients.

Previous work from our laboratory indicated that soluble TREM-1 is generated after LPS challenge or pathogen infection via limited proteolysis of membrane-anchored receptor by LPS-activated matrix metalloproteinases [9]. However, the low levels of TREM-1 expression on the cell surface of CF monocytes do not seem to be explained by proteolytic processing of the full-length receptor, as there are no significant differences in sTREM-1 levels found in CF patients and healthy volunteers. Thus, the clear disparity in TREM-1 levels on the monocyte surface between CF and controls cannot be explained by an exacerbated proteolytic attack secondary to pathogen infection in CF patients. In this regard, we notice that the kinetics of TREM-1 expression upon LPS stimulation was not significantly different in healthy volunteers and in the extra control group of COPD patients included in our study (see Table 4). Similar results have been recently reported by Radsak and co-workers [49]. Further, analysis of proinflammatory cytokines revealed that COPD monocytes are not locked in an endotoxin tolerance state (data not shown).

Both PGE_2_ and p65 have been described as positive regulators of TREM-1 expression [13], [40]. However, our results also seem to exclude a reduction in PGE_2_ levels and deficient activation of p65 as part of the molecular mechanism that leads to the observed tolerance state. On the other hand, the transcription factor PU.1 is known to block TREM-1 expression in murine macrophages [40]. In line with this finding, we have detected high levels of PU.1 in nuclei isolated from CF monocytes.

Currently, we can only speculate about the mechanisms that link defective CFTR functioning to TREM-1 down-regulation. One appealing hypothesis is that changes in the ion concentrations in CF patients induce PU.1 activation and subsequent TREM-1 down-regulation in their monocytes [40]. Interestingly, Xu and co-workers have reported that IL-1β and the related S100 calcium-binding proteins, calgranulin A and B (S100A8 and A9, respectively), are up-regulated in mice with CFTR deficiency [50]. In fact, elevated concentrations of these proteins, which have been alternatively termed CF antigen (CFAg), have long been detected in serum from CF patients [51], [52]. On the other hand, other authors have demonstrated that both IL-1β [53], [54] and S100A8 / A9 are induced upon PU.1 activation [55], [56]. Also along these lines, a recent study suggests a clear down-regulation of the transcriptional repressor, Foxp1, in *cftrΔ508* mice [57]; independent studies have established that this occurs in the presence of high levels of PU.1 [58]. Altogether, a likely pathway of monocyte inactivation in CF patients begins to emerge. Several lines of evidence indicate that transcription factor PU.1 is activated in CF models, perhaps in response to an elevated intracellular calcium concentration [59]. The transcription factor then accumulates at high levels in nuclei of human CF monocytes, ultimately resulting in *TREM-1* repression. The final result is a diminished expression of membrane-anchored TREM-1, which aborts the positive feed-back loop needed for mounting a strong response against invading pathogens.

In conclusion, our data suggest that circulating monocytes from CF patients are “locked” in an endotoxin tolerance state, which results at least in part from a notable down-regulation of TREM-1 expression. These findings open new avenues for the characterization and treatment of inflammatory responses in CF patients. Our results suggest that not only resident immune cells but also circulating monocytes are affected in CF patients, and are unable to respond properly to LPS challenge. We suggest that this information should be considered while developing new anti-infection therapies in CF patients.
